# The use and predictive performance of the Peninsula Health Falls Risk Assessment Tool (PH-FRAT) in 25 residential aged care facilities: a retrospective cohort study using routinely collected data

**DOI:** 10.1186/s12877-022-02973-0

**Published:** 2022-04-01

**Authors:** Nasir Wabe, Joyce Siette, Karla L. Seaman, Amy D. Nguyen, Magdalena Z. Raban, Jacqueline C. T. Close, Stephen R. Lord, Johanna I. Westbrook

**Affiliations:** 1 Centre for Health Systems and Safety Research, Australian Institute of Health Innovation, Macquarie University, Sydney, New South Wales Australia; 2 The MARCS Institute for Brain, Behaviour and Development, Western Sydney University, Sydney, Australia; 3 St Vincent’s Clinical School, UNSW Medicine, UNSW, Sydney, NSW Australia; 4 Neuroscience Research Australia, Sydney, New South Wales Australia; 5 School of Public Health and Community Medicine, University of New South Wales, Sydney, New South Wales Australia

**Keywords:** Accidental falls, Aged, Long term care facility, Home care, Predictive model, Fall risk assessment

## Abstract

**Background:**

The Peninsula Health Falls Risk Assessment Tool (PH-FRAT) is a validated and widely applied tool in residential aged care facilities (RACFs) in Australia. However, research regarding its use and predictive performance is limited. This study aimed to determine the use and performance of PH-FRAT in predicting falls in RACF residents.

**Methods:**

A retrospective cohort study using routinely-collected data from 25 RACFs in metropolitan Sydney, Australia from Jul 2014-Dec 2019. A total of 5888 residents aged ≥65 years who were assessed at least once using the PH-FRAT were included in the study. The PH-FRAT risk score ranges from 5 to 20 with a score > 14 indicating fallers and ≤ 14 non-fallers. The predictive performance of PH-FRAT was determined using metrics including area under receiver operating characteristics curve (AUROC), sensitivity, specificity, sensitivity_Event Rate(ER)_ and specificity_ER_.

**Results:**

A total of 27,696 falls were reported over 3,689,561 resident days (a crude incident rate of 7.5 falls /1000 resident days). A total of 38,931 PH-FRAT assessments were conducted with a median of 4 assessments per resident, a median of 43.8 days between assessments, and an overall median fall risk score of 14. Residents with multiple assessments had increased risk scores over time. The baseline PH-FRAT demonstrated a low AUROC of 0.57, sensitivity of 26.0% (sensitivity_ER_ 33.6%) and specificity of 88.8% (specificity_ER_ 82.0%). The follow-up PH-FRAT assessments increased sensitivity_ER_ values although the specificity_ER_ decreased. The performance of PH-FRAT improved using a lower risk score cut-off of 10 with AUROC of 0.61, sensitivity of 67.5% (sensitivity_ER_ 74.4%) and specificity of 55.2% (specificity_ER_ 45.6%).

**Conclusions:**

Although PH-FRAT is frequently used in RACFs, it demonstrated poor predictive performance raising concerns about its value. Introducing a lower PH-FRAT cut-off score of 10 marginally enhanced its predictive performance. Future research should focus on understanding the feasibility and accuracy of dynamic fall risk predictive tools, which may serve to better identify residents at risk of falls.

**Supplementary Information:**

The online version contains supplementary material available at 10.1186/s12877-022-02973-0.

## Introduction

Falls in older people are a common and challenging health problem causing significant morbidity, mortality and economic burden [1–3]. Approximately one in three people aged 65 years and over fall every year [4]. Globally, the age-standardised prevalence of falls is estimated to be over 5000 per 100,000 people [5]. According to the 2017 global burden of disease study from 195 countries, falls resulted in over half a million deaths, 16.7 million years of life lost, 19.3 million years lived with disability and 35.9 million disability-adjusted life years [5]. The occurrence and consequences of falls are even more concerning in residential aged care facilities (RACFs) [6] (also called care homes, nursing homes or long-term care). In Australia, people aged 65 years and over in RACFs are approximately five times more likely to experience a fall and six times more likely to experience fall-related injury compared to people of the same age in the community [4, 7]. In 2018/19, nearly 11% of permanent residents aged 65 years or over in RACFs were admitted to hospitals or visited emergency departments due to falls [8].

Falls risk assessment tools (FRATs) have been utilised in acute, subacute and aged care settings to help identify those at highest risk of falling [9]. FRATs provide fall risk profiles to predict the likelihood of future occurrences of falls and therefore can play a critical role in targeted fall prevention programs [10]. Several FRATs have been developed over the past two decades [9]. Examples of commonly used FRATs for older people in hospital or community settings include STRATIFY (St Thomas’s risk assessment tool in falling elderly inpatients) [11], Morse Fall Scale [12], Berg Balance Scale [13] and FROP-Com (falls risk for older people in the community) [14]. A recent systematic review identified fifteen FRATs suitable for use among older adults in RACFs, [15] including the Peninsula Health Falls Risk Assessment Tool (PH-FRAT) [16].

PH-FRAT, developed in 1999 by Peninsula Health in Victoria, Australia, is a validated and easy to use tool that can be used for both screening (early identification of individuals at risk of falls) and assessment (including identification of risk factors) and management strategies for reducing fall risk [17]. The tool was developed using a sample of 291 patients from a single site receiving subacute and residential aged care services. The original validation study reported a moderate predictive performance with a sensitivity of 58.4% and specificity of 90.1% [16]. Although the tool has been widely used in Australia [16, 18], there has been little further evidence of its effectiveness in predicting falls in settings. To the best of our knowledge, only one small study has evaluated its performance in RACFs [19] and there have been no published studies reporting its performance in routine clinical practice. Our aim was to conduct a large pragmatic study to understand the extent of routine use and the performance of PH-FRAT in predicting the occurrence of falls in 25 RACFs managed by one large aged care provider..

## Methods

### Study design and settings

We conducted a retrospective, longitudinal, cohort study using routinely collected de-identified aged care data from 1 July 2014 to 31 December 2019 extracted from 25 RACFs managed by a large not-for-profit aged care provider in New South Wales (NSW), Australia. We followed the REporting of studies Conducted using Observational Routinely-collected health Data (RECORD) statement [20] when writing this paper.

### Participants

**Fig. 1:**
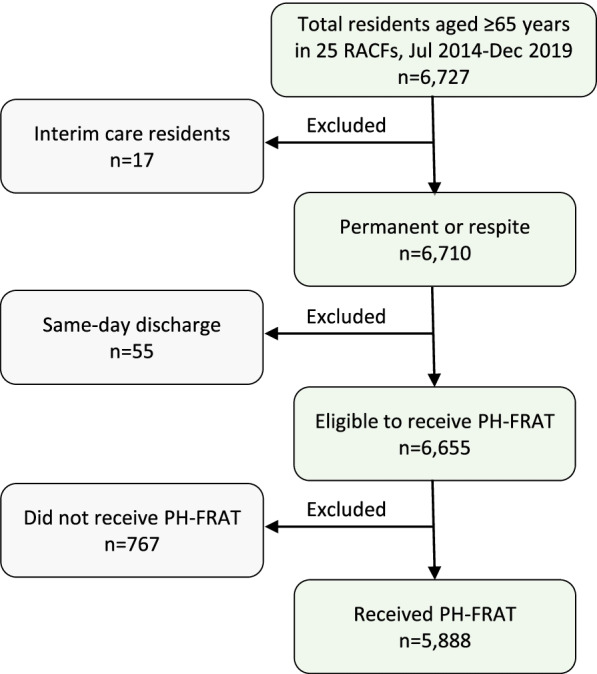
Participant selection flow chart. RACFs, Residential Aged Care; PH-FRAT, Peninsula Health Falls Risk Assessment Tool

The study population flow chart is shown in Fig. 1. The eligibility criteria included residents aged ≥65 years, who stayed at the facility for more than 24 h and had at least one PH-FRAT assessment. We excluded interim care (temporary stay) and same-day discharge (i.e. residents with a length of stay < 24 h at the facility) residents as these residents stayed in the facility for a short period of time and therefore had lower rates of falls and PH-FRAT assessment data relevant to this study.

### Data sources

The data used for this study were sourced from electronic health records used to collect clinical and care management data, data from PH-FRAT assessments and fall incident data. Twenty-five RACFs used the PH-FRAT tool [16] to assess residents’ falls risk. Assessments are conducted upon entry to facilities and over time to monitor a residents’ falls risk, identify risk factors and create a personalised management plans for high-risk residents. The PH-FRAT comprises three parts: fall risk status, risk factor checklist and action plan. For this study, we focused on the fall risk status data. Fall risk status is calculated by identifying whether four major fall risk factors (recent falls, medications, psychological status and cognitive status) were evident. The total risk score ranges from 5 to 20 with a score of > 14 indicating likely fallers and ≤ 14 non-fallers. A three-level fall risk classification of low (scores 5–11), medium (12–15) and high (16–20) has been used to identify and provide targeted fall preventive services to high-risk residents [16].

All residents’ falls were reported using a standardised incident form containing information on incident date and time, location of the incident, body region injured if there was an injury, and whether transfer to hospital was required. In addition to the fall risk and incident data, we extracted relevant data on socio-demographics (e.g. age, gender, country of birth), length of stay and clinical characterisitics (e.g. fall history at admission, health status).

### Statistical methods

Descriptive statistics including medians with inter-quartile ranges (IQR) were reported. We compared the characteristics of included and excluded participants using χ^2^ statistics for categorical variables and the Wilcoxon rank-sum test for continuous variables. To determine the predictive performance of the tool, the PH-FRAT scores were compared against the actual (observed) falls. The actual (observed) falls data were obtained from the facility incident form. We limited the follow-up period to the first six months after the PH-FRAT assessment as the tool was not designed for a long-term prediction. Observed falls were defined as the occurrence of any fall regardless of whether an injury was involved or hospitalisation required. Initially, we evaluated the performance of PH-FRAT using resident’s baseline risk status. Given PH-FRAT can be reapplied multiple times and its performance may change over time, we also reported the performance of the tool using the risk status at follow-up assessments**.** We present the results of the second to fifth applications of the tool. As we aimed to assess the performance of the tool in predicting falls, falls that occurred before the completion of the PH-FRAT assessment were excluded from the analysis. For instance, when evaluating the performance of the second PH-FRAT application against the occurrence of falls, only falls that occurred after the completion of the second PH-FRAT were included in the analysis.

The predictive performance of PH-FRAT was determined using commonly used performance metrics including sensitivity, specificity, positive predictive value (PPV), negative predictive value (NPV) and area under receiver operating characteristics (ROC) curve (AUROC) along with their 95% confidence intervals (CI). AUROC is an indicator of the discriminatory power of a given tool [21] (that is the ability of PH-FRAT to efficiently discriminate between fallers and non-fallers in this case). The AUROC values range from 0.5 to 1 with values from 0.5–0.6, 0.6–0.7, 0.7–0.8, 0.8–0.9 and 0.9–1.0 suggesting respectively poor, sufficient, good, very good and excellent discrimination [21]. We also report Youden’s index (sensitivity + specificity − 1), a point on ROC curves that is farthest away from the diagonal/reference line indicating optimal sensitivity and specificity of a given tool [21, 22]. Youden’s index ranges from 0 (no discrimination) to 1 (perfect discrimination) [21, 22].

We used two methods to calculate the performance measures including the standard and *event rate* (ER) methods. The formulas used to calculate these measures using both methods are presented in Supplementary Table 1. The *event rate* method was based on an approach proposed previously [23] and is the preferred method as it accounts for the recurrent nature of falls. The 95% CIs for sensitivity_ER_ and specificity_ER_ were determined using a bootstrapping technique as described by Haines et al. [23].

In addition to the original cut-off value of 14 to define fall risk status, we also determined a cut-off that best fitted our data. We used the highest Youden’s index_ER_ (a value on ROC curves associated with the highest sensitivity and specificity) to determine the optimal cut-off [22]. We report all performance measures of the new cut-point in a similar way as the original cut-point. A sub-group analysis by resident type (permanent vs respite) was also reported. All *p*-values were 2-tailed and alpha was set at *P* < 0.05. Analysis was conducted using Stata version 16 (StataCorp LP, College Station, TX).

## Results

### Participants

Of the total 6727 residents, 839 were excluded from the study (767 did not receive the tool, 55 had a same-day discharge and 17 were interim care residents). Of the 6655 eligible residents, 5888 (88.5%) received at least one PH-FRAT assessment (Fig. 1). Table 1 compares the baseline and follow-up characteristics of the included (*n* = 5888) and excluded (*n* = 839) participants. Except for age, all other characteristics were significantly different between the included and excluded participants. Included participants had a higher prevalence of comorbidities, were born in Australia, and died before the end of the study. The median age in the included participants was 86 (IQR 81–90) and 65.9% were female (Table 1).

**Table 1:** Comparison of included and excluded participants

	Included (***n*** = 5888)	Excluded (***n*** = 839)	***P***-value
Gender, n(%)			
Male	2006 (34.1)	316 (37.7)	0.038
Female	3882 (65.9)	522 (62.3)	
Age at admission, median (IQR)	86 (81–90)	86 (80–90)	0.352
Age at admission, mean (SD)	84.9 (7.7)	84.6 (8.2)	
Age category in year, n (%)			
65–74	617 (10.5)	107 (12.7)	0.215
75–84	1822 (30.9)	259 (30.9)	
85–94	2993 (50.8)	415 (49.5)	
≥ 95	456 (7.8)	58 (6.9)	
Country of birth, n (%)			
Australia	3737 (63.5)	437 (52.1)	< 0.001
Other country	2151 (36.5)	402 (47.9)	
Resident status at the end of the study^a^, n (%)			
Active	2390 (40.6)	591 (70.4)	< 0.001
Deceased	3498 (59.4)	248 (29.6)	
Resident type, n (%)			
Permanent admission^b^	4794 (81.4)	331 (39.5)	< 0.001
Respite admission only	1094 (18.6)	508 (60.5)	
Selected health status, n (%)			
Dementia	3044 (51.7)	234 (27.9)	< 0.001
Depression, mood and affective disorders	2479 (42.1)	177 (21.1)	< 0.001
Cognitive impairment	1980 (33.6)	178 (21.2)	< 0.001
Anxiety and stress-related disorders	1893 (32.2)	100 (11.9)	< 0.001
Cerebrovascular accident	1494 (25.4)	129 (15.4)	< 0.001
Diabetes mellitus	1331 (22.6)	159 (19.0)	0.017
Visual impairment	984 (16.7)	74 (8.8)	< 0.001
Delirium	593 (10.1)	33 (3.9)	< 0.001
Parkinson’s disease	442 (7.5)	40 (4.8)	0.004

χ2 was used to compare the categorical variables and the Wilcoxon rank-sum tests was used to compare the continuous variables respectively. ^a^The status of the 77 residents (same-day discharge or interim care) were recorded at the time of discharge or end of the follow-up period. ^b^Included some residents who had respite admission before permanent admission

### PH-FRAT utilisation

For the 5888 residents who received at least one PH-FRAT assessment, 38,931 assessments were performed with a median of 4 assessments per resident (IQR 2–8; range 1–107). For residents with multiple PH-FRAT assessments, the median time between assessments was 43.8 days (IQR 10.7–144.0). The overall median PH-FRAT risk score was 14 (IQR 11–16).

**Fig. 2:**
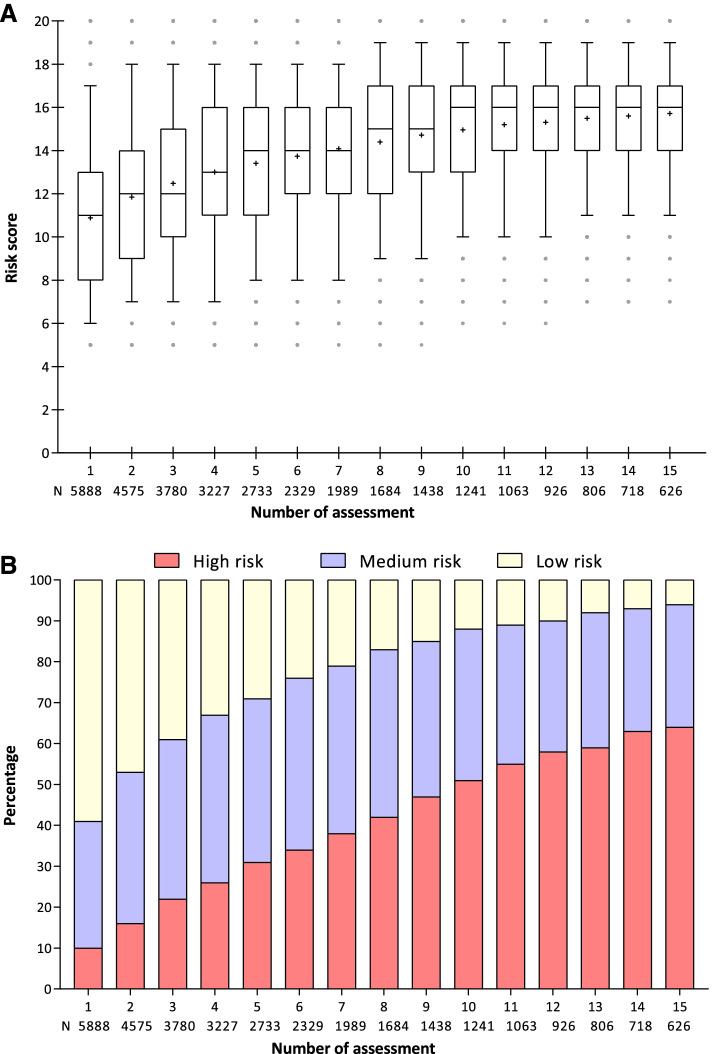
Trends in PH-FRAT risk scores (**A**) and risk groups (**B**) over time. Risk groups based on risk score: low (5–11), medium (12–16) and high (16–20)

Figure 2 shows the risk scores and risk groups for residents’ first and subsequent PH-FRAT assessments. We reported the first fifteen assessments for residents receiving PH-FRAT on multiple occasions. There was an upward trend in risk scores from first to the 10th assessment (increasing from median scores of 11 to 16) and then little change after the 10th assessment. The proportion of residents classified as high risk (risk score of 16–20) increased consistently over time from 10.0% at the first assessment to 63.7% at assessment fifteen (Fig. 2). It is important to note that having a fall in RACFs increases the subsequent PH-FRAT score as a history of ‘recent falls’ is one of the components of the PH-FRAT assessment [16]. For example, having a fall in the past 3 months while in a RACF automatically increases the subsequent PH-FRAT score by 8 points [16].

### PH-FRAT performance

A total of 27,696 falls were reported over 3,689,561 resident days (a crude incident rate of 7.5 falls/1000 resident days; 95% CI 7.4–7.6) for the 5888 residents during the entire follow-up period. Of the 27,696 fall incidents, 26,448 (95.5%) were reported after a baseline PH-FRAT assessment was completed. Sixty-two percent of residents (*n* = 3627) experienced at least one fall after the baseline PH-FRAT assessments. In the first 6 months, there were 7487 falls over 820,752 resident days (a crude incident rate of 9.1 falls/1000 resident days; 95% CI 8.9–9.3).

Table 2 presents the performance of baseline PH-FRAT assessments to accurately categorise residents as ‘fallers’ or ‘non fallers’ by comparing against the actual (observed) occurrence of falls after limiting the follow-up period to the first 6 months of PH-FRAT assessment. The ROC curve is shown in Supplementary Figure 1. Using a risk score cut-off of 14 (recommended by the original developer) [16], the AUROC was 0.57 indicating poor predictive performance. The tool had high specificity (88.8%; 95% CI 87.8–89.9) indicating the tool accurately predicted 88.8% of residents as unlikely to fall but had a low sensitivity (26.0%; 95% CI 24.3–27.8) in that it was only able to predict 26.0% of residents who were likely to fall. When the *event rate* method was utilised, the sensitivity_ER_ increased to 33.6% (95% CI 30.3–36.8) while maintaining a moderately high specificity_ER_ of 82.0% (95% CI 80.9–83.1).

**Table 2:** The performance of baseline PH-FRAT against the actual fall occurrence within the first six months of PH-FRAT assessment

	Risk score cut-off 14				Risk score cut-off 10			
PH-FRAT Predicted Falls	Observed Falls		No. of falls	Resident days	Observed Falls		No. of falls	Resident days
	Fallers	Non-fallers			Fallers	Non-fallers		
Fallers	627	388	2512	147,454	1626	1559	5570	446,080
Non-fallers	1782	3091	4975	673,298	783	1920	1917	374,672
Total	2409	3479	7487	820,752	2409	3479	7487	820,752
**Standard method**								
AUROC (95% CI)	0.57 (0.56–0.59)				0.61 (0.60–0.63)			
Sensitivity (95% CI)	26.0 (24.3–27.8)				67.5 (65.6–69.4)			
Specificity (95% CI)	88.8 (87.8–89.9)				55.2 (53.5–56.9)			
PPV (95% CI)	61.8 (58.7–64.8)				51.1 (49.3–52.8)			
NPV (95% CI)	63.4 (62.1–64.8)				71.0 (69.3–72.7)			
Youden’s index	0.148				0.227			
**Event rate method**								
Sensitivity_ER_ (95% CI)	33.6 (30.3–36.8)				74.4 (71.9–76.9)			
Specificity_ER_ (95% CI)	82.0 (80.9–83.1)				45.6 (44.2–47.1)			
Youden’s index_ER_	0.156				0.201			

*AUROC* Area Under Receiver Operating Characteristics curve, *PPV* Positive Predictive Value, *NPV* Negative Predictive Value. AUROC interpretation: poor (0.5–0.6), sufficient (0.6–0.7), good (0.7–0.8), very good (0.8–0.9) and excellent discrimination (0.9–1.0). Youden’s index ranges from 0 (no discrimination) to 1 (perfect discrimination)

By examining the ROC curve (Figure S1) we were able to identify the cut-off point at which PH-FRAT presents the optimal sensitivity and specificity. The highest Youden’s index was obtained at a risk score cut-off of 10 (a score of > 10 indicating fallers and ≤ 10 non-fallers). Using this cut-off, the tool had an AUROC of 0.61, the sensitivity improved significantly to 67.5% (95% CI 65.6–69.4) with a specificity of 55.2% (95% CI 53.5–56.9). When the *event rate* method was utilised, sensitivity further increased to 74.4% (95% CI 71.9–76.9) and specificity_ER_ dropped to 45.6% (95% CI 44.2–47.1) (Table 2).

**Fig. 3:**
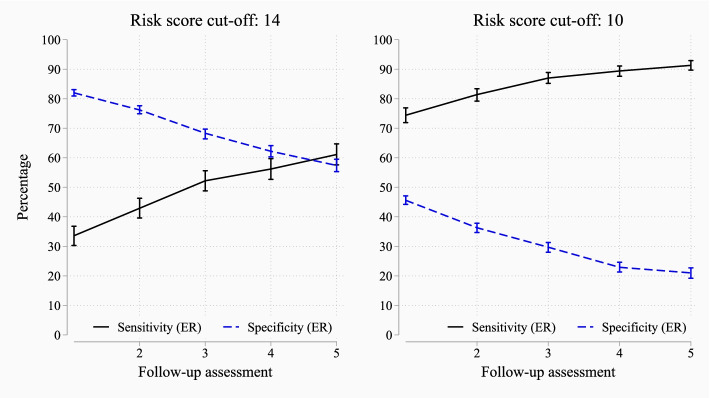
The performance of PH-FRAT against the actual fall occurrence at the second to the fifth follow-up assessments. ER, Event Rate. The error bars represent 95% CI

Figure 3 presents the sensitivity_ER_ and specificity_ER_ of PH-FRAT using the follow-up assessments (i.e. after residents have been reassessed on multiple occasions). The results from the second to the fifth assessments are presented. For both cut-off values, the sensitivity_ER_ improved with subsequent assessments, while the specificity_ER_ decreased. For instance, using the cut-off value of 14, the sensitivity_ER_ increased from 42.9% at the second assessment to 61.1% at the fifth assessment, while the specificity_ER_ decreased from 76.2 to 57.4% (Fig. 3).

### Sub-group analysis

A subgroup analysis by resident type showed no major difference in the performance of PH-FRAT by resident type. The AUROC for respite vs permanent residents was 0.55 vs 0.57 (using a cut-off of 14) and 0.61 for both groups (using a cut-off of 10). The performance of PH-FRAT was slightly better in respite compared to permanent residents when the *event rate* method was used (Youden’s index_ER_ of 0.224 vs 0.150 using a cut-off of 14 and 0.312 vs 0.191 using a cut-off of 10) (Table 3).

**Table 3:** The performance of baseline PH-FRAT assessments compared to predict actual fall occurrence for respite and permanent residents within the first six months of PH-FRAT assessment

	Risk score cut-off 14		Risk score cut-off 10	
	Permanent (***n*** = 4794)	Respite (***n*** = 1094)	Permanent (***n*** = 4794)	Respite (***n*** = 1094)
**Standard method**				
AUROC (95% CI)	0.57 (0.56–0.58)	0.55 (0.52–0.58)	0.61 (0.59–0.62)	0.61 (0.57–0.65)
Sensitivity (95% CI)	26.9 (25.1–28.8)	16.0 (11.1–21.9)	68.1 (66.1–70.1)	60.3 (53.1–67.2)
Specificity (95% CI)	87.0 (85.7–88.3)	94.0 (92.2–95.5)	53.0 (51.1–54.9)	61.4 (58.2–64.6)
PPV (95% CI)	66.1 (60.9–67.2)	36.5 (26.3–47.6)	55.5 (53.6–57.3)	25.2 (21.3–29.4)
NPV (95% CI)	58.1 (56.5–59.7)	83.8 (81.4–86.1)	65.9 (63.9–68.0)	87.8 (85–90.2)
Youden’s index	0.139	0.100	0.211	0.217
**Event rate method**				
Sensitivity_ER_ (95% CI)	33.7 (30.4–37.0)	30.8 (15.6–45.9)	74.6 (72.0–77.2)	71.3 (61.9–80.7)
Specificity_ER_ (95% CI)	81.3 (80.1–82.4)	91.6 (89.1–94.1)	44.5 (43.0–46.0)	59.9 (55.5–64.2)
Youden’s index_ER_	0.150	0.224	0.191	0.312

*AUROC* Area Under Receiver Operating Characteristics curve, *PPV* Positive Predictive Value, *NPV* Negative Predictive Value. AUROC interpretation: poor (0.5–0.6), sufficient (0.6–0.7), good (0.7–0.8), very good (0.8–0.9) and excellent discrimination (0.9–1.0). Youden’s index ranges from 0 (no discrimination) to 1 (perfect discrimination)

## Discussion

### Key findings

This study is one of very few studies to describe the utilisation pattern of PH-FRAT and determine its predictive performance against actual falls observed in RACFs in a large sample. We retrospectively evaluated the frequency of use and ability of the PH-FRAT risk assessment tool to predict falls among nearly 6000 residents in 25 RACFs. We found the PH-FRAT was frequently used with 89% of eligible residents receiving at least one assessment. However, the predictive performance of the tool was poor, accurately predicting a fall (within 6 months of PH-FRAT assessment) in only 33.6% of residents. With subsequent assessments, sensitivity improved but the specificity decreased. By changing the cut-off score by which the PH-FRAT categorises ‘fallers’ and ‘non-fallers’ from 14 to 10, the sensitivity and specificity of the PH-FRAT changed to 74 and 46% respectively.

### Interpretation and comparison with existing literature

The predictive performance PH-FRAT in our study was lower than that reported in the original validation study [16]. Stapleton et al. [16] reported a moderate predictive performance of the tool with a sensitivity of 58.4% (sensitivity_ER_ 68.8%), specificity of 90.1% (specificity_ER_ 70.2%) and Youden’s index of 0.49 (Youden’s index_ER_ 0.39). The main reason for the difference may be related to the dissimilar characteristics of our study population and that of the origin study [16]. In the original study (*n* = 291), only 20% of the patients were from nursing homes while the remainder were admitted for rehabilitation services (60%) or to hostels (20%) unlike in our study where all patients were from RACFs. Our study population was also older (mean age of 85.2 vs 79.9 years) with higher levels of comorbidities compared to the population used to develop the tool. It is likely that the original study overstated their predictive accuracy due to the retrospective validation design [24], a limitation recognised by the original developers [16].

External validation of FRATs beyond the original data is fundamental to establishing their generalizability. However, external validation is rarely performed, and in the domain of falls, only a small number of prediction models for community-dwelling older adults have been externally validated [25, 26], showing modest predictive accuracy [26–28]. Barker et al. [19] conducted a prospective external validation study to evaluate the psychometric properties (predictive, evaluative and discriminative validity) of four FRATs widely utilised in Australian RACFs including the PH-FRAT, Queensland Fall Risk Assessment Tool (QFRAT), Melbourne Fall Risk Assessment Tool (MFRAT), and the Falls Assessment Risk and Management Tool (FARAM). Although the study had a small sample consisting of 87 aged care residents, multiple sites including nursing homes and hostels were used. All tools exhibited poor psychometric properties. PH-FRAT had moderate sensitivity (52%), specificity (66%) and a Youden’s index (0.18) which was comparable with our findings when a risk score cut-off 10 was used. However, when the researchers compared the predictive accuracy of the tools against a single screening question “*has the resident fallen in the past 12 months?*”, all four tools performed no better than the screening question.

Our finding that the sensitivity_ER_ values increased while specificity_ER_ decreased with reapplications of the tool is consistent with the observation by Haines et al. [23] when evaluating the risk of falls among hospitalised patients (*n* = 316). Haines reported a dramatic increase in sensitivity_ER_ when a fall risk screening tool was reapplied [23]. This is likely to occur due to an increased risk of a resident falling and therefore being classified as true over time, which equates to increased sensitivity_SE_. As sensitivity (true positive rate) and specificity (true negative rate) are reciprocal to each other [29], the inverse relationship between sensitivity_ER_ and specificity_ER_ values over multiple assessments is not surprising. Additionally, given specificity values tend to decrease with an increasing prevalence of disease [30], a possible increase in the prevalence of falls over time might have contributed to the decrease in the specificity_ER_ values at follow-up times.

### Implications for clincal practice and recommendations for further research

PH-FRAT is one of the most frequently used tools in RACFs in Australia [15] and is currently adopted for inclusion in subacute fall prevention guidelines by the Australian Commission on Safety and Quality in Health Care [4]. Previous studies have shown its use to be highly feasible taking only 2–3 min to complete and it has demonstrated good inter-rater reliability [16, 19]. The high uptake of PH-FRAT in RACFs confirms its feasibility in routine clinical practice. However, PH-FRAT might need to be updated to reflect the current aged care population profile. Indeed, the poor predictive performance of PH-FRAT raises practical concerns about its utility and on whether it may be contributing to poor or untimely care decisions [31, 32]. This has safety implications as potentially high-risk residents eligible for specific fall prevention programs are likely to miss out on receiving the intervention due to incorrect risk profiling. We found that reducing the risk score cut-off point from 14 to 10 substantially improved the tool’s performance with a dramatic increase in the sensitivity_ER_ from 33.6 to 74%. We recommend using a revised cut-off score of 10 to define falls risk status – for example – a risk score of > 10 to indicate high risk and ≤ 10 low risk. For RACFs already using the tool, this change can be implemented easily given the current procedures already in place.

However, it is important to emphasise that most predictive tools, in general, do not perform well outside the original study population [33]. A study of 31 clinical prediction models that were externally validated, only 6 showed a comparable predictive performance in the validation studies [34]. This could be due to possible differences in health care practices and population charactistics [31]. Indeed, predictive factors relevant in the original population may no longer be applicable in another setting. For instance, of the items included in PH-FRAT that were predictive of falls in the original study, only fall history and psychological status predicted actual falls in a study by Barker et al. [19] whereas increasingly recognised risk factors for falls such as walking aids use and certain medical conditions (e.g. Parkinson’s disease) were not included in PH-FRAT [35].

Updating PH-FRAT through model recalibration by incorporating any new, relevant predictors may enhance the predictive performance of the tool. Although opinion varies regarding modifying the existing tool by incorporating new predictors among researchers [32], recalibration can help to optimise the predictive performance of a tool in a new setting [31].

Future research should also focus on understanding the feasibility and effectiveness of *dynamic fall risk prediction* models using routinely collected data which can reflect contemporary changes in residents’ risk factors. The existing FRATs used in RACFs [15] have been based on a *static prediction* using input variables collected at a single time point without incorporating the potential changes in the status of input variables over time. A model that incorporates all potentially useful information about input variables on an ongoing basis (dynamic prediction) could a play critical role in improving the prediction of falls. Dynamic predictions involve the use of real-time or near real-time data to enable up-to-date risk predictions. In long-term care settings such as RACFs risk factors for falls (e.g., medication utilisation) changes over time. However, electronic data containing relevant fall risk factors including both time-invariant (e.g. demographic) and time-varying (e.g. medication) factors are now collected as part of routine care in RACFsproviding unique opportunities to develop and test dynamic falls risk prediction tools [36, 37]. Several studies have identified that certain medications that are used for the treatment of conditions affecting cardiovascular (e.g., beta-blockers, diuretics) or central nervous systems (e.g., antipsychotics, sedatives) are known to increase the risk of falling [38–45]. As older people in RACFs are the primary users of these medications, it is important to utilise medication data as one of the main time-varying factors to obtain a robust and accurate dynamic prediction and monitoring of falls risk over time. Advanced statistical methods such as *joint models* [46], *landmark models* [47] and deep learning-based machine learning approaches [48] have previously been utilised to develop dynamic prediction models in other settings.

### Strengths and limitations

This is the first study to evaluate the use and performance PH-FRAT using routinely collected aged care data. The strength of our study lies in the methodology. Firstly, our study is a multi-centre study that involved a large sample of nearly 6000 residents from 25 RACFs. Secondly, unlike previous studies [16, 19], we utilised a longitudinal cohort design with long-term follow-up which allowed us to track residents over a combined period of greater than 2.5 million resident days. Routinely collected data has the added advantage of not being influenced by the study aims, minimal selection bias as a loss to follow-up or non-response is not an issue, and not subject to recall bias and differential misclassification [49]. Finally, in addition to reporting the performance metrics using the standard method, we utilised a modified approach based on event rate which serves a better indicator for recurrent events like falls [23].

The main limitation of this study was our focus on part 1 of the tool (risk score), which meant that the potential effect of subsequent fall prevention interventions was not accounted for in the current analysis. In addition to the risk score, PH-FRAT provides possible actions that can be implemented to prevent falls. For instance, residents that were predicted to be in a high-risk group at baseline might have received targeted fall prevention interventions and thus potentially decreased their risk of falling. This can confound the relationship between PH-FRAT and the likelihood of falls occurring. Our study was further restricted to RACFs in metropolitan areas and one aged care provider only, thus our findings may not be representative of all RACFs.

## Conclusion

In conclusion, although PH-FRAT is frequently used in RACFs, it demonstrated poor predictive performance against the occurrence of actual falls. This raises concerns about its utility and value and may be preventing some residents from gaining access to necessary fall prevention interventions. Reducing the PH-FRAT score to a lower cut-off value of 10 may optimise its predictive performance. Future research should also focus on understanding and improving the feasibility and effectiveness of dynamic fall risk predictive tools using routinely collected electronic aged care data to address the underlying limitation of static falls risk assessment tools.

## Supplementary Information

**Additional file 1: Table S1.** Measures of performance of PH-FRAT against actual fall occurrence.

**Additional file 2: Figure S1.** ROC curve for the baseline PH-FRAT in predicting falls in six month.

## Data Availability

The data that support the findings of this study are available from the aged care provider (Anglicare), but restrictions apply to the availability of these data, which were used under license for the current study, and so are not publicly available. Data are however available from the authors upon reasonable request and with permission of Anglicare.

## Abbreviations

AUROC: Area under receiver operating characteristics curve
CI: Confidence interval
ER: Event rate
FARAM: Falls Assessment Risk and Management Tool
FRATs: Falls risk assessment tools
FROP-Com: Falls risk for older people in the community
IQR: Inter-quartile ranges
MFRAT: Melbourne Fall Risk Assessment Tool
NPV: Negative predictive value
NSW: New South Wales
PH-FRAT: Peninsula Health Falls Risk Assessment Tool
PPV: Positive predictive value
QFRAT: Queensland Fall Risk Assessment Tool
RACFs: Residential aged care facilities
ROC: Receiver operating characteristics curve
STRATIFY: St Thomas’s risk assessment tool in falling elderly inpatients
