# Supplementary material for: The use and predictive performance of the Peninsula Health Falls Risk Assessment Tool (PH-FRAT) in 25 residential aged care facilities: a retrospective cohort study using routinely collected data
Source: BMC Geriatr. 2022 Apr 1;22:271. doi: 10.1186/s12877-022-02973-0 (PMC8973529; doi:10.1186/s12877-022-02973-0)
Supplement: Supplementary file 1 — Additional file 1: Table S1. Measures of performance of PH-FRAT against actual fall occurrence. [file 12877_2022_2973_MOESM1_ESM.docx]

**Supplementary Table**

**Table S1: Measures of performance of PH-FRAT against actual fall occurrence.**

|  | Observed Falls | | | No. of falls | Resident days |
| --- | --- | --- | --- | --- | --- |
| Predicted Falls (PH-FRAT) | Fallers | Non-fallers | Total |  |  |
| Fallers | A | C | A+C | N_A_ | RD_A_ |
| Non-fallers | B | D | B+D | N_B_ | RD_B_ |
| Total | A+B | C+D | A+B+C+D | N_A+B_ | RD_A+B_ |
| **Standard method** |  | | | | |
| Sensitivity | A/(A+B) | | | | |
| Specificity | D/C+D | | | | |
| Positive Predictive Value | A/A+C | | | | |
| Negative Predictive Value | D/B+D | | | | |
| Youden's Index | Sensitivity + Specificity -1 | | | | |
| **Event rate method** |  | | | | |
| Sensitivity_ER_ | N_A_/N_A+B_ | | | | |
| Specificity_ER_ | RD_B_/RD_A+B_ | | | | |
| Youden's Index_ER_ | Sensitivity_ER_ + Specificity_ER_ -1 | | | | |

ER, Event Rate.
