# Supplementary material for: The use and predictive performance of the Peninsula Health Falls Risk Assessment Tool (PH-FRAT) in 25 residential aged care facilities: a retrospective cohort study using routinely collected data
Source: BMC Geriatr. 2022 Apr 1;22:271. doi: 10.1186/s12877-022-02973-0 (PMC8973529; doi:10.1186/s12877-022-02973-0)
Supplement: Supplementary file 2 — Additional file 2: Figure S1. ROC curve for the baseline PH-FRAT in predicting falls in six month. [file 12877_2022_2973_MOESM2_ESM.pdf]

Figure S1: ROC curve for the baseline PH-FRAT in predicting falls in six months

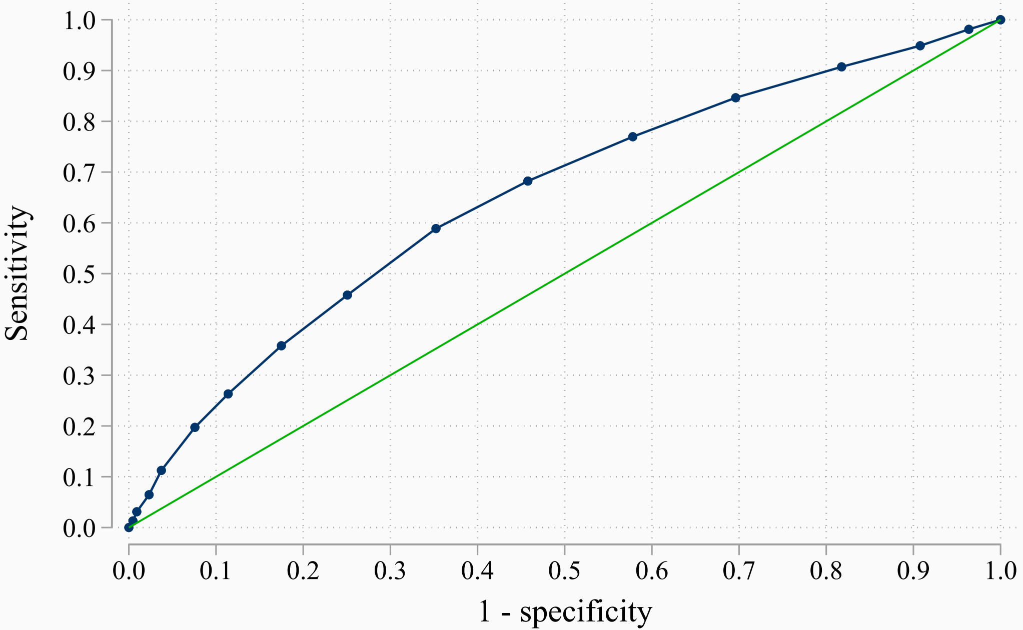

Area under ROC curve = 0.6497
